# Case report: maple syrup urine disease with a novel DBT gene mutation

**DOI:** 10.1186/s12887-019-1880-1

**Published:** 2019-12-13

**Authors:** Wei Feng, Jinfu Jia, Heyang Guan, Qing Tian

**Affiliations:** 1 Tianjin Medical University, Tianjin, China; 2 Chongqing Medical University, Chongqing, China; 3 Tianjin First Central Hospital, Tianjin, China

**Keywords:** Maple syrup urine disease, DBT gene mutation, Thiamine, Children

## Abstract

**Background:**

Maple syrup urine disease (MSUD) is a potentially life-threatening metabolic disorder caused by decreased activity of the branched-chain α-ketoacid dehydrogenase (BCKD) complex. Mutations in four genes (BCKDHA, BCKDHB, DLD and DBT) are associated with MSUD. Here, the presenting symptoms and clinical course of a case of MSUD with a novel DBT gene mutation are described.

**Case presentation:**

We describe an infant with MSUD with the DBT gene mutation who had drowsiness and poor appetite as well as abnormal findings upon head magnetic resonance imaging (MRI), plasma amino acid analysis and urine organic acid analysis. Genetic testing revealed that both parents had the heterozygous mutation c.1132C > T (p.378X) in chr1:100672078, and the patient had the homozygous mutations c.1132C > T (p.378X) in chr1:100672078. Once diagnosed with MSUD, the patient’s disease was controlled with a diet of BCAA-free enteral formula and thiamine.

**Conclusion:**

The mutation c.1132C > T (p.378X) is a novel DBT gene mutation that is associated with MSUD and always has mild clinical manifestations. After timely BCAA-free nutrition and supplementation with thiamine for the patient, the plasma levels of BCAAs reached a safe level, the abnormal range of the multiple intracranial abnormalities was significantly smaller than before, and the symptoms of drowsiness and poor appetite disappeared.

## Background

Maple syrup urine disease (MSUD) is a rare metabolic disorder of autosomal recessive inheritance caused by decreased activity of the branched-chain α-ketoacid dehydrogenase (BCKD) complex. The first cases of MSUD were described by Menkes et al. [1] in 1954. The incidence of MSUD is 1 in 185,000 births throughout the world, and the incidence rate may be higher in some ethnic and racial groups [2]. The deficiency of BCKD causes the corresponding branched-chain keto acids (BCKAs) formed by branched-chain amino acid (BCAA) transaminase to be unable to oxidize decarboxylic acid, resulting in the accumulation of BCAAs (including leucine, isoleucine and valine) and BCKAs. Within the brain, glutamate serves as a neurotransmitter within the central nervous system and plays important roles in brain development and cognitive functions. Disorders of BCAA metabolism can cause abnormalities in glutamate synthesis, leading to various neurological problems in patients. The accumulation of leucine is highly neurotoxic. Elevated levels of leucine can affect water homeostasis within the subcortical grey matter, causing swelling within the brain, altering nitrogen homeostasis, further depleting glutamate levels, and increasing oxidative stress [3]. Furthermore, the BCAAs and BCKAs that accumulate in MSUD trigger apoptosis in glial and neuronal cells in a dose- and time-dependent manner [4]. The accumulation of BCAAs and BCKAs induces ketoacidosis, developmental disturbances, neurological impairment and coma and may be fatal if untreated [5]. We analysed the clinical data of 1 case to improve the understanding of the disease and the level of diagnosis and treatment.

## Case presentation

### Clinical data and laboratory examinations

**Fig. 1:**
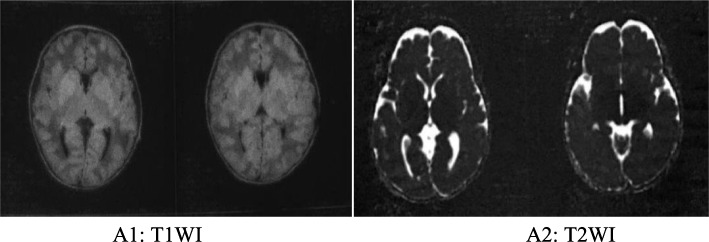
The MRI at the age of 14 days:DWI revealed that the cortical plate was slightly thin,the white matter area was relatively large, and the sulcus was slightly shallow. Symmetrical long T2 and DWI high-signal shadows were seen in the bilateral central gyrus, corona radiata, posterior limbs of internal capsules, thalamic region and cerebellar dentate nucleus

The male infant was the first child of consanguineous parents (the child’s father’s grandmother and his mother’s grandmother were sisters) from China, with a birth weight of 3200 g. There was no history of birth asphyxia. The boy had been breastfed. At the age of 11 days, he was admitted to the hospital due to drowsiness and poor appetite for 7 days. The family members had no similar disease. He had no obvious dysmorphic features. Cardiovascular, respiratory and abdominal examinations were normal. However, a peculiar, fruity urine odour was noted from the urine that was “reminiscent of decaying pears”. Plasma levels of lactate and glucose were normal, as were conventional liver tests and blood count. The abnormal clinical data and laboratory examinations were as follows: 1) 2 days after admission, he was diagnosed with MSUD based on plasma amino acid analysis, which showed elevated BCAAs, with leucine at 3281.5 μmol/L (normal range 57.16–246.95 μmol/L), isoleucine at 514 μmol/L (normal range 36.2–112.5 μmol/L) and valine at 613.28 μmol/L (normal range 46.16–231.70 μmol/L); 2) the levels of urine organic acid were measured with gas chromatography-mass spectrometry, and the patient demonstrated elevated 2-ketoisocaproic acid, 2-keto-3-methylvaleric acid and hydroxyisovaleric acid; 3) an electroencephalogram showed moderate abnormality in that bursts of 7 to 9 Hz spindle-like sharp waves of moderate amplitude were abundant in the frontocentral region bilaterally; 4) magnetic resonance imaging (MRI) of the head was performed on the third day of admission, and diffusion-weighted imaging (DWI) revealed that the cortical plate was slightly thin, the white matter area was relatively large, and the sulcus was slightly shallow; 5) symmetrical long T2 and DWI high-signal shadows were seen in the bilateral central gyrus, corona radiata, posterior limbs of the internal capsules, thalamic region and cerebellar dentate nucleus (Fig. 1).

### Genetic testing

**Fig. 2:**
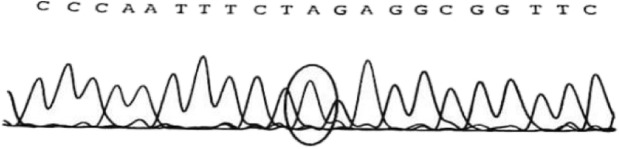
Genetic testing of patient: The homozygous mutation of c.1132C > T (p.378X) in chr1:100672078. The 1132th base of cDNA changed from C to T that caused the amino acid 378 (glutamine) to became a stop codon, leading to the early termination of protein translation. The homozygous mutation decreased activity of BCKD that causes the accumulation of BCAAs and BCKAs

**Fig. 3:**
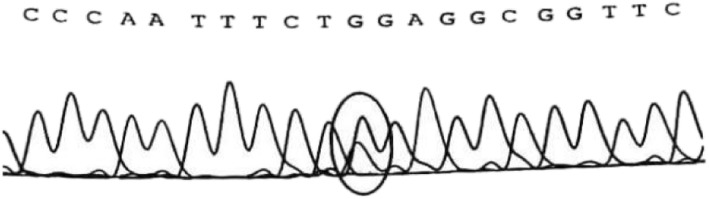
Genetic testing of the patient’s parents: Both the father and mother carried heterozygous mutation of c.1132C > T (p.378X) in chr1:100672078

Approximately 2 mL of the peripheral blood of the child and his parents was collected in tubes with ethylenediaminetetraacetic acid as an anticoagulant and sent to Beijing Jinhuai Medical Laboratory for the detection of DX-09_branched-chain amino acid metabolism disorder (suggested MSUD) by Agilent exon chip capture + high-throughput sequencing. The child had the homozygous mutation of c.1132C > T (p.378X) in chr1:100672078 (Fig. 2). Direct sequencing analysis of the parents revealed that both parents had the heterozygous mutation of c.1132C > T (p.378X) in chr1:100672078 (Fig. 3). It was suggested that the child had a malignant homozygous mutation in the DBT gene, and the father and mother were heterozygous carriers of the mutation. In addition, this mutation met the criteria for strong pathogenicity 1 (PS1) in the American Society of Medical Genetics and Genomics (ACMG) guidelines.

### Treatment and follow-up

**Fig. 4:**
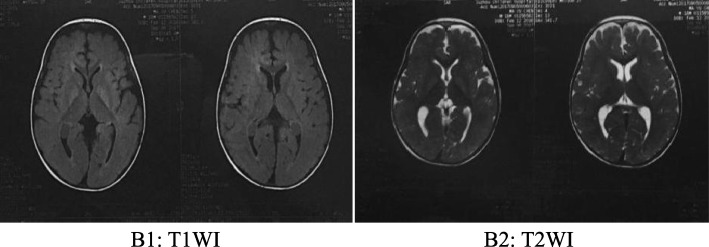
The MRI at the age of 2.5 years: The bilateral cerebral hemisphere of gray matter and white matter is well demarcated, and the symmetrical long T2 and DWI of central gyrus, corona radiata, posterior limbs of internal capsules, thalamic region and cerebellar dentate nucleus have improved than before.The abnormal signal of inner capsule basically disappeared.

Generally, the safe plasma levels of leucine are 75–1000 μmol/L, those for isoleucine are 200–400 μmol/L, and those for valine are 200–400 μmol/L in MSUD [6]. After admission, the patient was monitored with electrocardiography and was given intravenous fluid replenishment. Once diagnosed with MSUD, his disease was controlled with a diet of BCAA-free enteral formula, restricted protein intake, and thiamine. Before discharge, the metabolic parameters of BCAAs reached a safe level, with leucine at 883.75 μmol/L, isoleucine at 314.33 μmol/L and valine at 365.28 μmol/L. The boy was discharged from the hospital 7 days later, and the advice for children for supplemental thiamine and restricted dietary intake of BCAAs was given to the parent. After discharge, the plasma BCAA levels of the patient were strictly monitored once a month. At the age of 2.5 years, the levels of leucine and valine were 782.19 μmol/L and 202.32 μmol/L, respectively. A re-examination of the head MRI showed that the abnormal signal range of the multiple intracranial abnormalities was significantly smaller than that of the first examination (Fig. 4).

## Discussion and conclusions

On the basis of the activity of BCKD and clinical manifestations, most studies have classified MSUD into five forms: a classic form, intermediate form, intermittent form, thiamine-responsive form and fatty acid amide dehydrogenase deficiency form. The classic form is the most serious and common phenotype, which shows onset in early neonates with poor prognosis and can rapidly develop into respiratory failure, coma and even death. The clinical onset of MSUD usually occurs within the first week after birth but lacks specific symptoms. The standard treatment of MSUD is mainly a special diet and thiamine intake, while patients with severe MSUD need dialysis and plasma exchange. BCAA-free enteral formula plays an essential role in restoring and maintaining metabolic homeostasis in MSUD, and patients should avoid the ingestion of BCAA-containing exogenous protein and liquid to prevent catabolism and the accumulation of endogenous BCAAs and BCKAs [7]. Thiamine pyrophosphate (TPP) is a co-factor for the multi-subunit enzyme BCKD, and thiamine supplementation is an adjunctive therapy to be considered in the treatment regime for MSUD. Patients with MSUD for whom supplemental thiamine provides increased dietary BCAA tolerance (or decreased plasma BCAAs on constant dietary intake) appear to be individuals with some residual BCKD activity [8], especially those with DBT gene mutations [9]. In our study, the boy was admitted to the hospital due to drowsiness and poor appetite at the age of 11 days. The clinical symptoms, examination indexes and head MRI of the child were significantly improved with BCAA-free enteral formula and thiamine diet. Based on the clinical presentation, progression of the disease and effectivity of thiamine, the patient might be classified as having a thiamine-responsive form of MSUD.

To date, at least 4 genes (BCKDHA, BCKDHB, DLD and DBT) have been reported to be the causative gene for MSUD [2]. According to the mutation types of the BCKDHA, BCKDHB, DBT and DLD genes, MSUD can also be divided into four genotypes: IA, IB, II and III. Among them, BCKDHA and BCKDHB mutations are the most common, and DLD gene mutations are rarely reported. Furthermore, there have been 88 BCKDHA, 85 BCKDHB, 21 DLD and 71 DBT mutations listed in the Human Gene Mutation Database (HGMD) [10]. Mitsubuchi et al. [11] considered that type IA and IB tended to be of the classic MSUD type and that all thiamine-responsive MSUDs are type II, with patients that have mild clinical manifestations [8]. This child suffering from a DBT gene mutation, with mild clinical manifestations, improved obviously after timely supplementation with thiamine, which is consistent with the above view. In this case, the child had a homozygous mutation of the DBT gene, c.1132C > T (p.378X), and both parents had the heterozygous mutation of c.1132C > T (p.378X), which has not yet been reported. The grandmothers of the father and mother were sisters, and the c.1132C > T mutation of the DBT gene was familial. However, the parents of the child denied that there was a similar disease in the family, which provided new thinking about the study of this disease.

MSUD is a disorder of branched-chain amino acid metabolism, with BCAAs and BCKAs accumulating in the blood and intracranially, and BCKAs can be excreted in the urine. Therefore, blood tandem mass spectrometry, urinary organic acid analysis and head MRI are helpful for early diagnosis. Blood tandem mass spectrometry always showed elevations of leucine, isoleucine and valine, and leucine was more significant. Schadewaldt et al. [12] proposed that the elevation of isoleucine is more specific for the diagnosis of MSUD, but the determination of isoleucine has not been widely used. In the acute phase, the accumulation of BCAAs and BCKAs affected the tricarboxylic acid cycle, thereby interfering with the energy metabolism of brain cells, leading to the dysfunction of the Na^+^/K^+^ ATP pump and diffuse cerebral oedema [13]. In the chronic phase, cavernous degeneration and myelin sheath formation disorder occurred in white matter due to the accumulation of BCAAs and BCKAs. The symmetrical central gyrus, basal ganglia, thalamus, dentate nucleus, and cerebral foot of the brain were damaged [14]. Therefore, blood tandem mass spectrometry can detect an increase in branched-chain amino acids before the symptoms of encephalopathy and craniographic changes occur in patients with MSUD [15]. In this case, the clinical manifestations and related tests and examinations were very important for us to make a timely diagnoses of MSUD. Timely diagnosis is beneficial for early intervention to improve the prognosis.

## Conclusion

In view of the clinical data of the patient, prompt diagnosis and treatment were essential to improve prognosis. Blood tandem mass spectrometry, urinary organic acid analysis and head MRI were crucial for the timely diagnosis. Furthermore, we identified a novel mutation of the DBT gene (c.1132C > T (p.378X)), and BCAA-free nutrition and a thiamine diet were effective to improve the blood level of BCAAs. This case provides an important reference for the diagnosis and treatment of MSUD.

## Data Availability

All data supporting the findings of this article are included in the manuscript.

## Abbreviations

BCAAs: Branched-chain amino acids
BCKAs: Branched-chain keto acids
BCKD: Branched-chain a-keto acid dehydrogenase
MSUD: Maple syrup urine disease
